# Diphtheria in Chewing-Gum

**Published:** 1890-10

**Authors:** 


### DIPHTHERIA IN CHEWING-GUM.

A contemporary thus calls attention to the possible spreading of diphtheria through chewing-gum :

"The practice of chewing gum has become very wide spread. It is not a very elegant habit ; to many it is positively repulsive ; and there are sources of danger, too, that should not be overlooked. A case in point was related to us a few days ago. Diphtheria broke out in a family in East Des Moines. After the child had recovered, the clothing and all the exposed articles fully disinfected, the parents, with the convalescent child, visited some relatives in the country. The indispensable chewing-gum, like Satan, went also—in the mouth of the little child. Prompted by generosity, it allowed its country cousins—two children—to chew also the gum previously chewed by the visiting child. In three or four days, without any other known source of infection than the chewing-gum, the two children were simultaneously stricken down with diphtheria in a most serious form. It would be hard to imagine a more successful mode of propagation—distributing the disease. It would be a great deal safer not to chew the stuff at all, but if it must be done to satisfy the demands of a weak head and a depraved appetite, our advice is, don't 'swap' gum to chew any body else's gum, nor allow any body else to chew yours."

